# Assessing the Three Attentional Networks and Vigilance in the Adolescence Stages

**DOI:** 10.3390/brainsci11040503

**Published:** 2021-04-16

**Authors:** Jasmine Giovannoli, Diana Martella, Maria Casagrande

**Affiliations:** 1 Dipartimento di Psicologia, Università degli Studi di Roma “La Sapienza”, 00185 Roma, Italy; 2 Instituto de Estudios Sociales y Humanísticos, Universidad Autónoma de Chile, Santiago de Chile 7500000, Chile; diana.martella@uautonoma.cl; 3 Dipartimento di Psicologia Dinamica, Clinica e Salute, Università degli Studi di Roma “La Sapienza”, 00185 Roma, Italy

**Keywords:** attention, alerting, orienting, executive control, ANTI-V, adolescents

## Abstract

Attention involves three functionally and neuroanatomically distinct neural networks: alerting, orienting, and executive control. This study aimed to assess the attentional networks and vigilance in adolescents aged between 10 and 19 years using the attentional network test for interaction and vigilance (ANTI-V). One hundred and eighty-two adolescents divided into three groups (early adolescents, middle adolescents, late adolescents) participated in the study. The results indicate that after age 15, adolescents adopt a more conservative response strategy and increase the monitoring of self-errors. All the attentional networks seem to continue to develop during the age range considered in this study (10–19 y). Performance improved from early adolescence to middle adolescence and began to stabilize in late adolescence. Moreover, a low level of vigilance seems to harm alerting and orienting abilities.

## 1. Introduction

Attention includes a series of processes through which individuals locate information in their consciousness and update their preexisting knowledge. According to Posner and Petersen’s neurocognitive model [1,2], attention involves three neural networks: alerting, orienting, and executive control. The alerting network aims to achieve (phasic alerting) and maintain (tonic alerting or vigilance) a general state of activation of the cognitive systems. The orienting network supports the ability to focus on and select specific information. The executive control network manages the ability to solve conflictual information. Fan et al. [3] developed the attentional networks test (ANT), combining the cued reaction time [4] and the flanker paradigm [5] to assess the efficiency of the three attentional networks. The ANT allows rapid and simultaneous evaluation of the three attentional networks, and it was used in different research contexts and different populations such as children [6,7,8], adults [9,10,11,12], older adults [13], and also clinical populations [14,15,16]. The classic version of the ANT use the same variable (visual cue) to measure alerting and orienting, preventing the assessment of the interaction between these two systems; moreover, the spatial cue is 100% predictive (i.e., cue and target appear always in the same location); therefore, it does not allow evaluation of the reorienting of attention due to invalid cues. Callejas et al. [17] designed a new version of this task to overcome these limits. In the attentional network test for interaction (ANTI), the double cue was replaced with an alerting tone (warning tone) presented in 50% of the trials; further, the percentage of predictivity of the spatial cue was manipulated, introducing 50% of valid trials and 50% of invalid trials. This new structure of the task allows us to independently assess the three networks and their interactions.

Vigilance (or tonic alertness) is the ability to sustain performance over a long period and detect a rare but critical event [18]. It is supported by top-down or endogenous processes driven by internal goals and enables one to maintain and focus attention voluntarily [19]. Vigilance consists of an executive component that allows the detection of an infrequent target and inhibits frequent responses. It is an arousal component necessary to achieve and sustain fast reactions to stimuli without much control (i.e., no alternative response). Tasks that evaluate the former component are the continuous performance test (CPT; Ref. [20]) and the Mackworth clock test (MCT; Ref. [21]). Tasks that assess the latter are the psychomotor vigilance test (PVT; Ref. [22]) and the sustained attention to response task (SART; Ref. [23]). Another way to study vigilance is to manipulate vigilance by using experimental paradigms of total sleep deprivation [24] and prolonged wakefulness [25,26].

The most used vigilance indices are the number of errors and omissions, and according to the signal detection theory, the d′ index (sensitivity index, i.e., the ability to distinguish between target and non-target stimuli), and the β index (an indicator of the bias in the response). These variables are indicators of overall performance, while it would be more useful to evaluate performance over time, using, for example, individual variability in response time [27].

Initial versions of the ANT did not include a direct measure of vigilance. Therefore, tonic alertness was assessed using indirect indices that were only moderately correlated with direct measures [28]. In particular, vigilance was measured through global reaction times (RTs) and global accuracy (ACC) [28], mean RTs in no-cue trials [29], the slope of change in RTs or standard error (SE) of RTs between blocks [30,31,32], and mean RTs in the last block minus mean RTs in the first block [33].

To overcome these limits, Roca et al. [28] developed a modified version of ANTI introducing a direct measure of vigilance. More precisely, the ANTI-vigilance (ANTI-V) measures the executive component of vigilance, requiring the participant to recognize infrequent stimuli while he/she is performing the main task.

The attentional networks follow different developmental trajectories. Some authors argue that phasic alertness development occurs from early childhood to late childhood [34,35,36]. Other studies claim that phasic alertness development would continue between 8.5 and 26.7 years [37] and that there is a negative correlation between phasic alertness and age [8,37]. Regarding the orienting ability, some authors propose that development reaches stability during middle childhood [8,34,35,36], while Boen et al. [37] assert that this ability would continue to develop from late childhood to young adulthood. Finally, executive control would develop through early adolescence to reach stability in late adolescence [37].

Few studies investigated the development of vigilance in the transition period from childhood to adolescence. Several authors agree that sustained attention develops consistently and rapidly between the ages of 5 and 10 years (see [38]), but it is still unclear what happens in the next period. Two longitudinal studies that investigated vigilance using the CPT [39,40] showed different results. Fortenbaugh et al. [39] observed a linear improvement of vigilance between ages 10 and 16, finding that between 10 and 14 years, there is a period characterized by the rapid development of skills marked by a less cautious response strategy is used and characterized by faster RTs.

Moreover, between 14 and 17 years, teenagers can change in response strategy by adopting a more conservative approach (reduced β, slower RTs) and an increase in error monitoring. Rebok et al. [40] showed a nonlinear trajectory: between the ages of 8 and 10 years, the omissions seem to decrease, while between 10 and 13 years, there is no substantial change. Another study [41] also pointed to a nonlinear trajectory of vigilance development between ages 7 and 10, while Lewis et al. [42] argued that no substantial changes occur between ages 8–9 and 10–11. Finally, Mcavinue et al. [43] indicated a U-shaped developmental trend in vigilance abilities, characterized by low performance in childhood and adolescence, a plateau in adulthood, and a decrease in the elderly. According to Morandini et al. [19], vigilance development is characterized by a rapid improvement throughout childhood and adolescence and a plateau in young adulthood with a peak in the mid-40s. These vigilance abilities tend to decline in late adulthood gradually.

Moreover, vigilance seems to affect phasic alertness and executive functions [44]. In particular, the benefit of a warning signal (phasic alertness) seems to be more intense in participants with low levels of vigilance, whereas it would give less advantage when participants had high vigilance. Furthermore, a higher sensitivity d′ (i.e., increased vigilance) corresponds to a lower interference of incongruent distractors (ACC) (i.e., increased inhibition response).

The use of ANTI-V in adolescence could provide a more global view of attention systems and vigilance, including their interactions in this developmental step. This study aims to analyze how attention and vigilance develop during adolescence and verify whether different levels of vigilance impact the other attentional networks. To our knowledge, no study used ANTI-V with typically developing children or adolescents. In contrast, it was used with adolescents with psychiatric diseases [15], young adults [44,45,46], elderly people [47], and clinical populations [16,48].

## 2. Materials and Methods

### 2.1. Participants

One hundred and eighty-two adolescents took part in the study. Participants were divided into three groups based on the WHO indication of stages of adolescence [49]: 58 early adolescents (EA; age range: 10–14 y; mean age: 12.78; females: 40), 87 middle adolescents (MA; age range: 15–17 y; mean age: 15.95; females: 33), 37 late adolescents (LA; age range: 18–19 y; mean age: 18.46, females: 18). All of them had normal or corrected to normal vision. The protocol was approved by the Ethics Committee of the Department of Dynamic and Clinical Psychology of the University of Rome “La Sapienza” (Protocol number: 0000636-20/05/2019), and all participants signed the informed consent.

### 2.2. Apparatus

The experiment was programmed and displayed by E-Prime software [50] on an Intel Core i5 PC and displayed on a 17-inch color screen. The participant’s responses were recorded through a standard keyboard, and the warning tone was presented through headphones.

### 2.3. Attentional Network Test for Interaction—Vigilance (ANTI-V)

**Figure 1:**
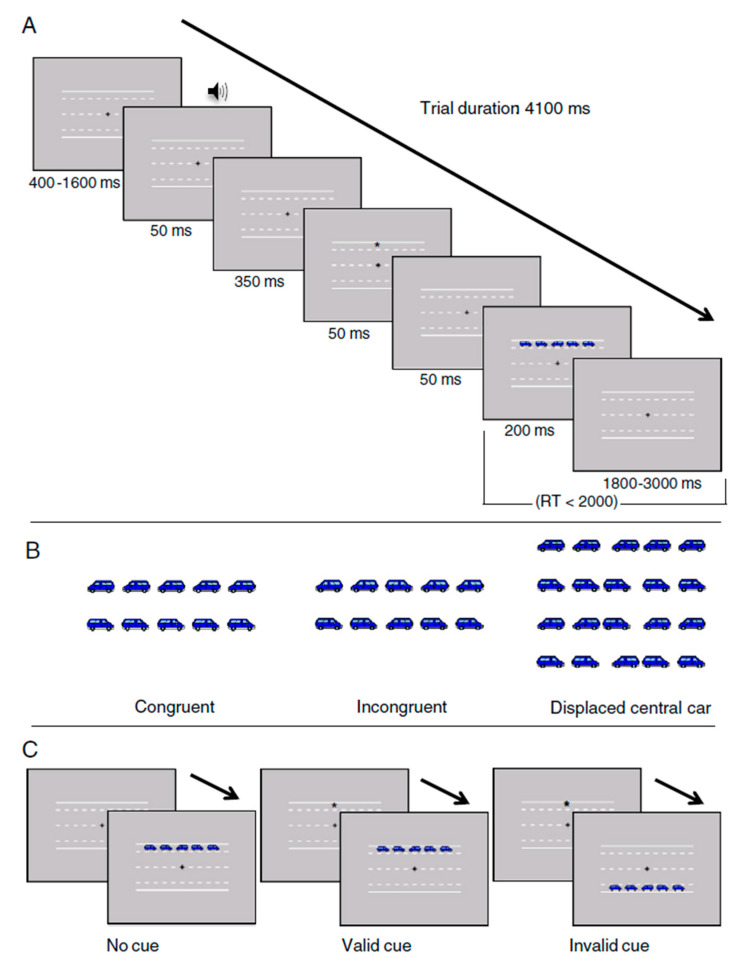
Procedure and stimuli of the ANTI-V. (**A**) Schematic representation of the procedure. (**B**) The target stimuli. (**C**) The visual cue conditions.

The ANTI-V [28,45] is a computerized task designed to assess alerting, orienting, executive attention, and their interactions; furthermore, it allows evaluating vigilance (for a detailed description of both the stimuli and the procedure, see Roca et al. [28]). The sequence of events in each trial is illustrated in Figure 1. The following stimuli were presented: a black fixation cross, a black asterisk, a warning tone, and a row of five cars pointing either left or right. The background was grey, and a two-lane road with two parking lanes was represented in the center of the screen. The central target car and its flankers appeared on one of the two parking lanes, above or below the fixation cross. Each trial began with a fixation period of variable duration (400–1600 ms). A 50 ms auditory warning signal was presented in half of the trials 500 ms before the target car was shown (warning tone condition) or not presented (no warning tone condition). Next, after another fixation period of 350 ms, an asterisk (orienting visual cue) was presented for 50 ms in the same location as the forthcoming target central car (valid visual cue condition), in the opposite position (invalid visual cue condition), or was preceded by no asterisk (no visual cue condition). Then for 200 ms, the target (central car) and its flankers were presented. Flanker cars could point to the same direction of the target (congruent condition) in half of the trials or the opposite direction of the target (incongruent condition) in the other half.

The task was composed of 4 blocks of 64 trials each (48 trials for the usual ANTI conditions and 16 vigilance trials with the displaced central target condition). In the first block (practice), feedback on accuracy was provided. A pause followed the first block, and there were no more rest periods until the end of the task.

The task required indicating the direction of the central car by pressing “c” (for left), “m” (for right), and spacebar (displaced central car). The instructions given were similar to those used by Roca et al. [28]). The task was presented as a game where participants worked in a Centre for Traffic Management and studied the drivers’ parking habits. Participants had to pay attention to the central car while fixing the central cross during the entire task. They were instructed to respond as fast and accurately as possible by pressing “c” (for left) or “m” (for right) on the keyboard. Finally, in 25% of the trials, the central target car was significantly displaced to the left or right. These trials allow assessing vigilance. In these trials, the distance of the central target car was manipulated, being either centered or significantly displaced (i.e., appearing closer to one of the near flanker cars). Additionally, the vertical and horizontal location of each car was slightly changed in each trial, adding a random variability (±4 pixels) to make it more difficult to distinguish between the centered and the displaced target car. The participants were encouraged to identify these infrequent stimuli by pressing an alternative response key (spacebar) and ignoring the direction of the central car in these trials. A period of 2000 ms was allowed for responses. The background road and the fixation point remained present until the end of the experiment. The entire session lasted approximately 30 min.

### 2.4. Data Analyses

Mean RTs of correct responses were filtered, disregarding the trials with extreme values that were higher or lower than the mean ± 2.5 SD per participant. The mean accuracy was higher than 89% (range: 78.12–95.95%). A Group (EA, MA, LA) × warning (warning, no-warning) × cue (valid, invalid, no-cue) × executive control (congruent, incongruent) mixed-design ANOVA was performed on the mean RTs of the correct responses, the mean percentage of accuracy, and the mean SD of RTs of the correct responses. Different attentional network scores were computed as a subtraction from specific average conditions: (a) alerting effect: no-warning minus warning conditions; (b) orienting effect: invalid minus valid conditions; (c) executive control: incongruent minus congruent conditions; (d) costs: invalid trials minus no-cue trials, and (e) benefits: no-cue trials minus valid trials. One-way ANOVAs on alerting, orienting, executive control, costs, and benefits effects were conducted to estimate the efficiency of each attentional system. The signal detection theory (SDT) indexes of sensitivity (d′) and response bias (β) were computed from hits (proportion of correct spacebar responses to infrequent targets) and false alarms (proportion of incorrect spacebar responses) to evaluate performance in the vigilance task. When the proportion of hits or false alarms was 0 or 1, those values were, respectively, substituted by 0.01 and 0.99 to obtain an appropriate approximation of the SDT indexes. Moreover, vigilance trials calculated the number of errors and omissions, the mean RTs, and the SD of RTs of correct responses. Vigilance performance indexes and attentional network scores were submitted to one-way ANOVAs with the Group (EA, MA, LA) as the between factor. Planned comparisons were used to analyze the main effects of the task and the interactions. An α value of 0.05 was used to establish statistical significance for all analyses. Data were analyzed using Statistica (Statsoft, Inc., Tulsa, OK, USA) v. 10.

### 2.5. Power Analysis

An a priori power analysis was conducted to calculate an adequate sample size for testing our hypotheses using a 3 (group) × 2 (warning) × 2 (executive control) × 3 (cue) repeated-measures design. Setting α at 0.05, power (1 − β) at 0.95, and expecting a correlation of ρ = 0.50 between repeated measures and a medium effect size of Cohen׳s f = 0.25, the power analysis (G*Power 3.1.9.4) [51] indicated that a sample size of at least 27 participants per group would yield an adequate power to detect a medium effect size. These results, thus, confirmed that the present study has enough statistical power to test our hypotheses.

## 3. Results

Table 1 shows the mean reaction times for correct responses and the percentage of accuracy for each condition and group.

### 3.1. Reaction Times

The analysis of variance showed significant main effects of warning (*F*_1,179_ = 96.47; *p* < 0.001; *η*^2^ = 0.35), executive control (*F*_1,179_ = 393.33; *p* < 0.001; *η*^2^ = 0.69), and cue (*F*_2,358_ = 114.01; *p* < 0.001; *η*^2^ = 0.39). Participants were faster when a warning tone was provided and in the congruent trials. Furthermore, participants were faster in valid than invalid trials (*F*_1,179_ = 193.40; *p* < 0.01; *η*^2^ = 0.52) and no-cue trials (*F*_1,179_ = 23.50; *p* < 0.01; *η*^2^ = 0.12). Participants were faster in no-cue than in invalid trials (*F*_1,179_ = 109.89; *p* < 0.01; *η*^2^ = 0.38).

**Figure 2:**
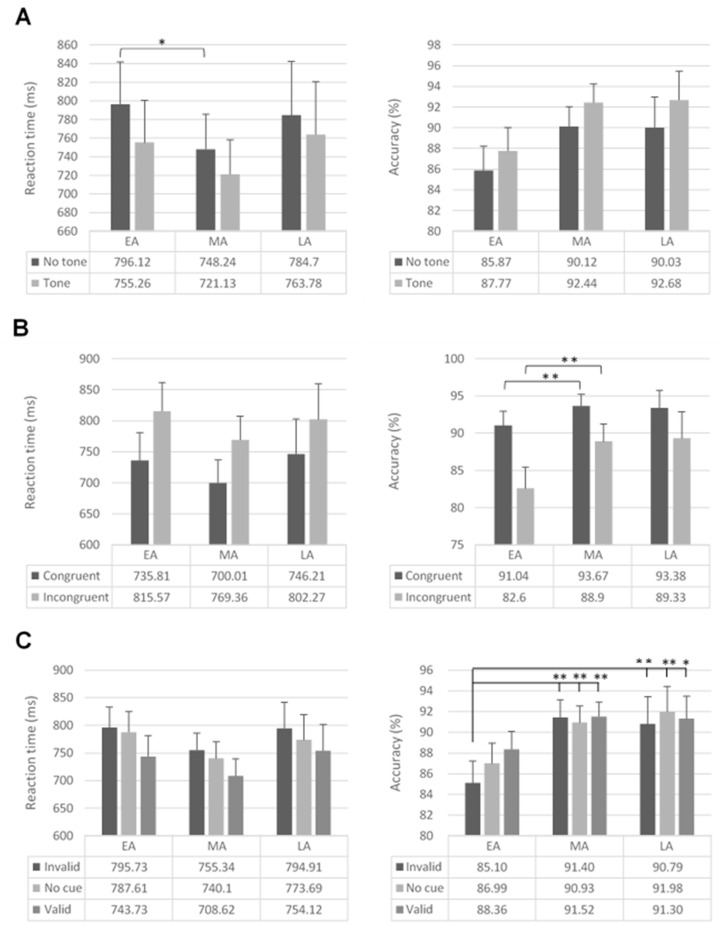
Mean reaction times (**left**) and percentage of accuracy (**right**) in the three groups of participants (early adolescents, middle adolescents, late adolescents) for (**A**) warning condition, (**B**) executive control condition, and (**C**) cue condition. Error bars represent standard errors. * *p* < 0.05; ** *p* < 0.01.

Furthermore, the group × warning (*F*_2,179_ = 3.62; *p* = 0.029; *η*^2^ = 0.04; Figure 2) and group × executive control (*F*_2,179_ = 3.32; *p* = 0.038; *η*^2^ = 0.04; Figure 2) interactions were significant. Specifically, considering warning condition, EA were slower than MA in no-warning trials (*F*_1,179_ = 3.92; *p* = 0.049; *η*^2^ = 0.02). Regarding executive control, EA were marginally slower than MA in incongruent trials (*F*_1,179_ = 3.61; *p* = 0.059; *η*^2^ = 0.02).

Considering cue, all participants were faster in valid trials than invalid (EA: *F*_1,179_ = 86.89; *p* < 0.001; *η*^2^ = 0.33; MA: *F*_1,179_ = 105.22; *p* < 0.001; *η*^2^ = 0.37; LA: *F*_1,179_ = 34.12; *p* < 0.001; *η*^2^ = 0.16) and no-cue trials (EA: *F*_1,179_ = 75.93; *p* < 0.001; *η*^2^ = 0.30; MA: *F*_1,179_ = 58.63; *p* < 0.001; *η*^2^ = 0.25; LA: *F*_1,179_ = 9.64; *p* < 0.002; *η*^2^ = 0.05). Moreover, MA and LA were faster in no-cue than invalid trials (MA: *F*_1,179_ = 13.32; *p* < 0.001; *η*^2^ = 0.07; LA: *F*_1,179_ = 10.99; *p* < 0.001; *η*^2^ = 0.06).

The group × cue interaction (*F*_4,358_ = 2.16; *p* = 0.073; *η*^2^ = 0.02) was marginally significant; all the other interactions were not significant (*F* < 2.30).

### 3.2. Accuracy Analysis

The main effects of group (*F*_2,179_ = 8.73; *p* < 0.001; *η*^2^ = 0.09), warning (*F*_1,179_ = 41.78; *p* < 0.001; *η*^2^ = 0.19), executive control (*F*_1,179_ = 117.51; *p* < 0.001; *η*^2^ = 0.40), and cue (*F*_2,358_ = 5.16; *p* = 0.006; *η*^2^ = 0.03) were significant. EA were less accurate than both MA (F_1,179_ = 15.23; *p* < 0.001; *η*^2^ = 0.08) and LA (*F*_1,179_ = 10.21; *p* = 0.002; *η*^2^ = 0.05). Furthermore, participants were more accurate in warning condition, when the cue was valid and the flanker was congruent.

The group × executive control (*F*_2,179_ = 6.71; *p* = 0.001; *η*^2^ = 0.07; see Figure 2) and group × cue (*F*_4,358_ = 4.07; *p* = 0.003; *η*^2^ = 0.04; Figure 2) interactions were significant.

Considering executive control, EA were less accurate than MA in congruent (*F*_1,179_ = 6.94; *p* = 0.009; *η*^2^ = 0.04) and incongruent trials (*F*_1,179_ = 17.46; *p* < 0.001; *η*^2^ = 0.09). EA were less accurate than LA in incongruent trials (*F*_1,179_ = 12.93; *p* < 0.001; *η*^2^ = 0.07), while EA were marginally less accurate in congruent trials (*F*_1,179_ = 3.59; *p* = 0.060; *η*^2^ = 0.02).

Regarding cue, EA had lower accuracy than MA (invalid: *F*_1,179_ = 20.99; *p* < 0.001; *η*^2^ = 0.10; no cue: *F*_1,179_ = 9.78; *p* = 0.002; *η*^2^ = 0.05; valid: *F*_1,179_ = 8.08; *p* = 0.005; *η*^2^ = 0.04) and LA (invalid: *F*_1,179_ = 11.14; *p* = 0.001; *η*^2^ = 0.06; no cue: *F*_1,179_ = 10.17; *p* = 0.002; *η*^2^ = 0.06; valid: *F*_1,179_ = 4.53; *p* = 0.035; *η*^2^ = 0.02).

Furthermore, the executive control × cue interaction (*F*_2,358_ = 5.95; *p* = 0.003; *η*^2^ = 0.03) revealed that in all cue conditions, participants were less accurate in incongruent trials than in congruent trials (invalid cue: *F*_1,179_ = 81.27; *p* < 0.001; *η*^2^ = 0.31; no cue: *F*_1,179_ = 75.26; *p* < 0.001; *η*^2^ = 0.30; valid cue: *F*_1,179_ = 45.18; *p* < 0.001; *η*^2^ = 0.20). Besides, when the trials were incongruent, participants were less accurate in invalid than no-cue (*F*_1,179_ = 4.30; *p* = 0.039; *η*^2^ = 0.02) and valid trials (*F*_1,179_ = 12.75; *p* < 0.001; *η*^2^ = 0.07). Participants were also less accurate in no-cue trials than valid trials (*F*_1,179_ = 4.07; *p* = 0.045; *η*^2^ = 0.02).

All the other interactions were not significant (*F* < 2.50).

### 3.3. Attentional Effects

#### 3.3.1. Reaction Times

One-way ANOVAs revealed a significant effect of group for alerting (*F*_2,179_ = 3.62; *p* = 0.029; *η*^2^ = 0.04) and executive control (*F*_2,179_ = 3.32; *p* = 0.038; *η*^2^ = 0.04). Considering alerting, EA had lower alerting ability than MA (*F*_1,179_ = 4.49; *p* = 0.003; *η*^2^ = 0.02) and LA (*F*_1,179_ = 6.12; *p* = 0.014; *η*^2^ = 0.03). Regarding executive control, EA had lower executive control than LA (*F*_1,179_ = 6.61; *p* = 0.011; *η*^2^ = 0.04).

Attentional benefit effect (*F*_2,179_ = 4.67; *p* = 0.010; *η*^2^ = 0.05) revealed that EA showed marginally lower benefits than MA (*F*_1,179_ = 3.64; *p* = 0.058; *η*^2^ = 0.02) and lower benefits than LA (*F*_1,179_ = 9.07; *p* = 0.002; *η*^2^ = 0.05).

**Figure 3:**
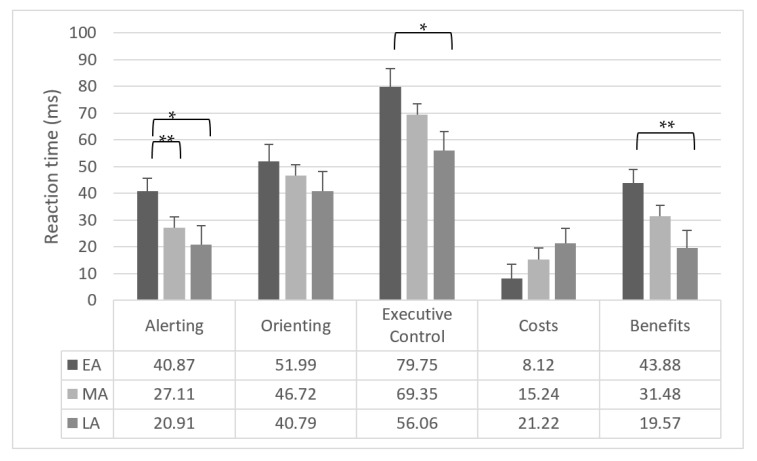
Mean reaction times of attentional effects: alerting, orienting, executive control, costs, and benefits. * *p* = 0.01; ** *p* < 0.01.

Orienting and attentional cost were not different among groups (*p* > 0.05). Figure 3 reported the attentional effects for the three groups of participants.

#### 3.3.2. Accuracy

One-way ANOVAs revealed a significant effect of group for orienting (*F*_2,179_ = 6.12; *p* = 0.003; *η*^2^ = 0.06) and executive control (*F*_2,179_ = 6.71; *p* = 0.001; *η*^2^ = 0.07).

EA showed lower orienting ability than MA (*F*_1,179_ = 11.47; *p* < 0.001; *η*^2^ = 0.06) and LA (*F*_1,179_ = 5.75; *p* = 0.017; *η*^2^ = 0.03).

Considering executive control, EA showed a higher interference than MA (*F*_1,179_ = 10.34; *p* = 0.001; *η*^2^ = 0.05) and LA (*F*_1,179_ = 9.56; *p* = 0.002; *η*^2^ = 0.05).

The attentional cost effect (*F*_2,179_ = 4.06; *p* = 0.019; *η*^2^ = 0.04) revealed that EA showed higher costs than MA (*F*_1,179_ = 7.53; *p* = 0.007; *η*^2^ = 0.04).

Alerting effect (*F* < 1) and attentional benefit (*p* > 0.05) were not different among groups.

### 3.4. Standard Deviation of RTs

The analysis of variance showed significant main effects of group (*F*_2,179_ = 3.18; *p* = 0.044; *η*^2^ = 0.03), warning (*F*_1,179_ = 17.99; *p* < 0.001; *η*^2^ = 0.09), and executive control (*F*_1,179_ = 26.74; *p* < 0.001; *η*^2^ = 0.13).

EA showed higher response variability than MA (*F*_1,179_ = 6.33; *p* = 0.013; *η*^2^ = 0.03). All participants showed higher variability in no-warning conditions and incongruent trials. All the other effects were not significant (*F* < 1.42).

### 3.5. Vigilance Analysis

One-way ANOVAs revealed a significant effect of group for hits (*F*_2,179_ = 9.42; *p* < 0.001; *η*^2^ = 0.09), d′ (*F*_2,179_ = 10.69; *p* < 0.001; *η*^2^ = 0.11), and percentage of errors (*F*_2,179_ = 8.40; *p* < 0.001; *η*^2^ = 0.09).

EA showed lower hits and d′ than MA (hits: *F*_1,179_ = 13.08; *p* < 0.001; *η*^2^ = 0.07; d′: *F*_1,179_ = 14.84; *p* < 0.001; *η*^2^ = 0.08) and LA (hits: *F*_1,179_ = 14.82; *p* < 0.001; *η*^2^ = 0.08; d′: *F*_1,179_ = 16.81; *p* < 0.001; *η*^2^ = 0.09).

Furthermore, EA made more errors than MA (*F*_1,179_ = 11.22; *p* < 0.001; *η*^2^ = 0.06) and LA (*F*_1,179_ = 13.59; *p* < 0.001; *η*^2^ = 0.07).

The effect of group for omissions (*F*_2,179_ = 2.87; *p* = 0.059; *η*^2^ = 0.03) revealed that EA made more omissions than both MA (*F*_1,179_ = 4.21; *p* = 0.042; *η*^2^ = 0.02) and LA (*F*_1,179_ = 4.30; *p* = 0.040; *η*^2^ = 0.02).

**Figure 4:**
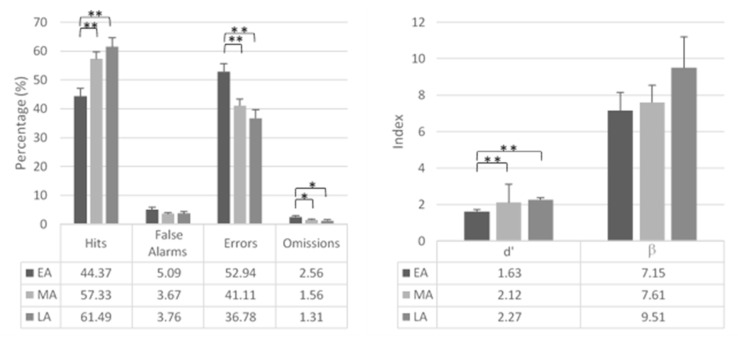
Mean vigilance indexes. (**Left**) Percentage of hits, false alarms, errors, and omissions. (**Right**) d′ and β indexes. * *p* < 0.05; ** *p* < 0.01.

Percentage of false alarms (*p* > 0.05) and β (*F* < 1) did not differ between groups. Figure 4 reports the vigilance indices for the three groups of adolescents.

The effect of group for mean RTs in vigilance blocks was marginally significant (*F*_2,179_ = 2.93; *p* = 0.056; *η*^2^ = 0.03). EA were slower than MA (*F*_1,179_ = 5.63; *p* = 0.019; *η*^2^ = 0.03). Mean SD of RTs in vigilance blocks did not differ between groups (*F* < 1).

Table 2 shows the vigilance measures for each group.

### 3.6. Correlations

Pearson’s correlations were used to assess the relationship between vigilance indexes, attention network scores, and the other attentional measures (see Table 3).

For RT data, the hits and d′ index were positively correlated with mean RTs, d′ was negatively correlated with alerting and orienting. False alarms were positively correlated with alerting and mean SD of RTs. β index was negatively correlated with mean RTs, alerting, executive control, and mean SD of RTs.

For accuracy data, hits and d′ were positively correlated with mean accuracy and orienting; moreover, d′ was positively correlated with executive control. False alarms were negatively correlated with mean accuracy and executive control, while β index was positively correlated with mean accuracy and executive control.

Hits and d′ were also negatively correlated with the percentage of errors and omissions.

### 3.7. Speed-Accuracy Trade-Off Effect

Within-network correlations between accuracy and RTs were used to examine the speed-accuracy trade-off effect according to age groups. Alerting effect RT of EA (*r* = −0.26, *p* = 0.046) and LA (*r* = −0.48, *p* = 0.003) was negatively correlated with mean accuracy. Mean RTs of MA (*r* = 0.32, *p* = 0.002) and LA (*r* = 0.39, *p* = 0.017) were positively correlated with orienting effect ACC. No other correlation was significant.

## 4. Discussion

Adolescence is a critical period characterized by the physical, social, and psychological development that determines the transition from childhood to adulthood [49]. Several studies investigated the development of attention [8,52] and vigilance [38,39]; however, only a few included participants who were in the transition phase from childhood to adolescence [35,36,37].

The present study highlights no significant differences between the groups in overall RTs, while the accuracy and intraindividual variability (mean SD) results indicate that performance improves with age. In both the ANTI trials and the vigilance trials, the early adolescents were less accurate than the other two groups (middle adolescents, late adolescents). This result seems to support the hypothesis that a strategic shift occurs after age 15, leading to the adoption of a more conservative response strategy and increased monitoring of self-errors [39].

Furthermore, the early adolescents achieved a lower d′ index than the other two groups confirming a greater difficulty in performing the task and distinguishing the target from the non-target that could indicate a poor vigilance. Moreover, early adolescents omitted more responses than the other two groups. Contrary to expectation [39], early adolescents were slower than the middle adolescents’ group in the vigilance trials. This result and the absence of significant differences between the groups in overall RTs could be due to the complexity of the task.

Both alerting and orienting abilities seem to continue to develop during the age range considered in this study (10–19 y). Performance improved from early adolescence to middle adolescence and began to stabilize late.

The level of vigilance seems to affect attentional networks [44]. An inverse relationship emerges between the vigilance (d′) and alerting (RT) effect. The warning tone would seem to have a greater effect on the group with lower vigilance (early adolescents). In the presence of a warning tone, the early adolescents improve their performance by 41 ms compared to the middle adolescents, which improves it by 27 ms, and the late adolescents by 21 ms. The effect of the warning tone seems to compensate for the poor vigilance ability partially. Orienting ability would also appear to be affected by the vigilance level. The results suggest that lower levels of vigilance seem to be associated with a lower orienting ability (ACC). Early adolescents appear to be less accurate than middle adolescents and late adolescents across all conditions and had higher attentional costs than middle adolescents. The results also indicate that early adolescents would benefit less from a valid visual cue than middle adolescents and late adolescents by showing slower RTs than the other groups.

Consistent with previous studies [37], executive control appears to develop through the early adolescence and stabilizes in late adolescence. Specifically, early adolescents show greater conflict than middle adolescents and late adolescents. Furthermore, lower sensitivity (d′) seems to be associated with larger interference of incongruent distractors in the accuracy [44].

No significant differences emerged between the groups regarding the β index, but it was negatively correlated with the mean reaction times and positively correlated with mean accuracy. These significant correlations would indicate that performing the task more slowly allowed the adoption of a more liberal response bias, and conversely, the use of a conservative response bias approach results in greater accuracy. No significant differences emerged between the groups about the percentage of false alarms. A positive correlation emerged between false alarms and the alerting effect (RT). This result would seem to suggest that a higher activation state may lead to the commission of more errors. Indeed, a higher state of alertness increases the speed of response but reduces the control functions [17].

## 5. Limitations

Future studies should include children, young adults, and elderly people in order to study the evolution of attentional networks across the lifespan. It would be necessary to conduct longitudinal studies to limit the effect of intraindividual variability.

Another limitation of the study could be the use of a test for adults, which has never been used in adolescents before. The complexity of the task could not have highlighted significant differences between the groups. However, it was previously used with adolescents’ psychiatric population [15]. Furthermore, we previously used the ANTI-V with children with ADHD [53,54]. According to previous data, we believe that adolescents could complete this task. Nevertheless, it would be useful to use easier tasks. Recently, a modified version of ANTI-V, the ANTI-Vea [18], was developed and found to be easier than the classic version by Roca et al. [28]. Furthermore, this version allows for assessment of the executive and the arousal component of vigilance. Another possibility would be to adapt an easier version of ANTI (e.g., the ANTI-Fruit; [12]) by adding a direct vigilance measure.

## 6. Conclusions

The present experiment is the first to examine the development and the interactions among attention networks using the ANTI-V in adolescents aged 10 through 19.

To summarize, adolescents’ performance improves with age. The number of errors and omissions decreases, and the ability to distinguish the target from the non-target increases. Moreover, all the attentional networks seem to develop during the first stages of adolescence and tend to stabilize during late adolescence.

The results indicate the importance of assessing the level of vigilance when studying attentional networks. Indeed, a low level of vigilance seems to harm alerting and orienting abilities. More studies should adopt experimental tasks such as the ANTI-V that include a direct measure of vigilance, since indirect indices were only moderately correlated with direct measures.

Furthermore, as already suggested by Boen et al. [37], future studies should include individual variability measures, which would seem to be a useful indicator to understand the development of attention better.

## Figures and Tables

**Table 1:** Mean reaction times (in milliseconds) and percentage of accuracy for each experimental condition in the three groups of participants.

			Early Adolescents (*n* = 58)		Middle Adolescents (*n* = 87)		Late Adolescents (*n* = 37)	
			RTs	Accuracy	RTs	Accuracy	RTs	Accuracy
No warning	Congruent	Valid	725.70 (140.14)	90.62 (9.46)	691.74 (141.98)	92.31 (6.92)	742.37 (165.55)	92.40 (11.33)
		Invalid	766.26 (147.16)	90.09 (11.41)	719.93 (135.93)	93.10 (6.81)	772.76 (186.44)	92.90 (7.53)
		No Cue	785.37 (153.56)	89.76 (10.90)	734.95 (155.42)	92.60 (6.14)	765.62 (147.33)	92.40 (7.24)
	Incongruent	Valid	797.60 (159.82)	84.37 (12.40)	758.58 (150.94)	89.08 (8.90)	785.06 (182.10)	88.68 (8.57)
		Invalid	856.61 (168.75)	78.12 (16.27)	798.26 (147.84)	86.92 (11.30)	822.79 (130.41)	87.33 (10.67)
		No Cue	845.22 (167.57)	82.22 (14.38)	785.99 (138.75)	86.71 (9.28)	819.58 (143.56)	86.49 (11.65)
Warning	Congruent	Valid	679.39 (154.53)	92.46 (8.58)	659.83 (141.95)	93.96 (6.63)	714.95 (153.55)	93.41 (6.58)
		Invalid	740.21 (149.78)	90.95 (8.77)	711.51 (144.15)	95.26 (5.13)	754.95 (164.77)	93.24 (9.01)
		No Cue	717.96 (131.95)	92.35 (9.22)	682.14 (139.25)	94.76 (6.66)	726.62 (150.75)	95.95 (4.93)
	Incongruent	Valid	772.25 (171.49)	85.99 (14.35)	724.34 (142.76)	90.73 (7.37)	774.08 (151.66)	90.71 (9.39)
		Invalid	819.84 (152.60)	81.25 (17.36)	791.66 (140.69)	90.30 (9.57)	829.15 (150.68)	89.70 (10.12)
		No Cue	801.89 (171.43)	83.62 (17.71)	757.34 (144.07)	89.65 (9.90)	782.95 (151.90)	93.07 (7.62)

**Table 2:** Mean reaction times (in milliseconds) and percentage of accuracy for each age group.

	Early Adolescents (*n* = 58)	Middle Adolescents (*n* = 87)	Late Adolescents (*n* = 37)
Hits (%)	44.37 (20.93)	57.33 (22.10)	61.49 (18.96)
False alarms (%)	5.09 (5.78)	3.67 (3.00)	3.76 (3.73)
Errors (%)	52.94 (20.52)	41.11 (22.03)	36.78 (18.19)
Omissions (%)	2.53 (3.38)	1.56 (2.89)	1.31 (1.67)
d′	1.63 (0.81)	2.12 (0.72)	2.27 (0.69)
β	7.15 (7.65)	7.61 (8.69)	9.51 (10.27)
Mean RTs (Vigilance block)	975.30 (177.19)	920.76 (115.29)	930.43 (100.12)
Mean SD of RTs (Vigilance block)	184.64 (66.17)	177.93 (58.22)	174.78 (49.77)

**Table 3:** Correlations between the signal detection theory indexes (hits, false alarms, d′, and β) and the attention networks scores, the mean reaction time, and the standard deviation of RTs (ANTI-blocks and vigilance blocks), the mean accuracy (ANTI-blocks), errors, and omissions (vigilance blocks).

	Hits	False Alarms	d′	β
ANTI Blocks				
Reaction time				
Alerting	−0.14 *p* = 0.070	0.17 *p* = 0.020	−0.25 *p* = 0.001	−0.17 *p* = 0.024
Orienting	−0.14 *p* = 0.070	−12 *p* = 0.112	−0.17 *p* = 0.025	−0.07 *p* = 0.355
Executive Control	−0.03 *p* = 0.715	0.09 *p* = 0.221	−0.11 *p* = 0.158	−0.16 *p* = 0.030
Mean RTs	0.33 *p* < 0.001	0.13 *p* = 0.087	0.18 *p* < 0.019	−0.19 *p* = 0.012
Mean SD of RTs	0.11 *p* = 0.144	0.19 *p* = 0.010	−0.04 *p* = 0.629	−0.21 *p* = 0.005
Accuracy				
Alerting	0.02 *p* = 0.767	0.002 *p* = 0.978	0.03 *p* = 0.643	0.005 *p* = 0.950
Orienting	0.24 *p* = 0.001	−0.14 *p* = 0.067	0.24 *p* = 0.001	−0.02 *p* = 0.800
Executive Control	0.02 *p* = 0.778	−0.54 *p* < 0.001	0.29 *p* < 0.001	0.32 *p* < 0.001
Mean ACC	0.24 *p* = 0.001	−0.82 *p* < 0.001	0.63 *p* < 0.001	0.41 *p* < 0.001
Vigilance blocks				
Mean RTs	−0.08 *p* = 0.257	−0.07 *p* = 0.370	−0.04 *p* = 0.576	0.03 *p* = 0.733
Mean SD of RTs	−0.01 *p* = 0.915	0.07 *p* = 0.375	−0.01 *p* = 0.921	0.03 *p* = 0.674
Errors	−0.99 *p* < 0.001	−0.05 *p* = 0.496	−0.79 *p* < 0.001	0.08 *p* = 0.294
Omissions	−0.18 *p* = 0.015	−10 *p* = 0.202	−0.19 *p* = 0.012	0.01 *p* = 0.939

## Data Availability

The data presented in this study are available in the Open Science Framework repository, https://osf.io/ujhwq/ (accessed on 1 April 2021)
